# Feed Insects as a Reservoir of Granadaene-Producing Lactococci

**DOI:** 10.3389/fmicb.2022.848490

**Published:** 2022-05-09

**Authors:** Vera Neuzil-Bunesova, Alejandro Ramirez Garcia, Nikol Modrackova, Marie Makovska, Monika Sabolova, Cathrin Spröer, Boyke Bunk, Jochen Blom, Clarissa Schwab

**Affiliations:** ^1^Department of Microbiology, Nutrition and Dietetics, Czech University of Life Sciences Prague, Prague, Czechia; ^2^Laboratory of Food Biotechnology, Institute of Food, Nutrition and Health, ETH Zürich, Zurich, Switzerland; ^3^Leibniz Institute DSMZ-German Collection of Microorganisms and Cell Cultures GmbH, Braunschweig, Germany; ^4^Bioinformatics and Systems Biology, University Giessen, Giessen, Germany; ^5^Department of Biological and Chemical Engineering, Aarhus University, Aarhus, Denmark

**Keywords:** orange pigment, *Lactococcus garvieae*, *Lactococcus petauri*, *Lactococcus formosensis*, insects, monkeys, granadaene

## Abstract

Insects are a component of the diet of different animal species and have been suggested as the major source of human dietary protein for the future. However, insects are also carriers of potentially pathogenic microbes that constitute a risk to food and feed safety. In this study, we reported the occurrence of a hemolytic orange pigmented producing phenotype of *Lactococcus garvieae/petauri/formosensis* in the fecal microbiota of golden lion tamarins (*Leontopithecus rosalia*) and feed larvae (*Zophobas atratus*). Feed insects were identified as a regular source of *L. garvieae/petauri/formosensis* based on a reanalysis of available 16S rRNA gene libraries. Pan-genome analysis suggested the existence of four clusters within the *L. garvieae/petauri/formosensis* group. The presence of *cyl* cluster indicated that some strains of the *L. garvieae/petauri/formosensis* group produced a pigment similar to granadaene, an orange cytotoxic lipid produced by group B streptococci, including *Streptococcus agalactiae*. Pigment production by *L. garvieae/petauri/formosensis* strains was dependent on the presence of the fermentable sugars, with no pigment being observed at pH <4.7. The addition of buffering compounds or arginine, which can be metabolized to ammonium, restored pigment formation. In addition, pigment formation might be related to the source of peptone. These data suggest that edible insects are a possible source of granadaene-producing lactococci, which can be considered a pathogenic risk with zoonotic potential.

## Introduction

Insects are a natural nutrient source of different animal species (Verbeke et al., 2015; Gasco et al., 2019). Moreover, insects are a dietary component of certain Asian and African countries. Globally, there is a search for alternative sources of protein to replace or substitute meat, and insects are an excellent source of essential nutrients, minerals, vitamins, and proteins (Raheem et al., 2019). This has led to considerable interest in edible and feed insects, such as crickets, grasshoppers, ants, and various beetle grubs, including mealworms, darkling beetle larvae, and caterpillars (Garofalo et al., 2019; Govorushko, 2019). Due to their short life cycles when reared and farmed, insects are considered a sustainable (Raheem et al., 2019), land-efficient alternative to animal-sourced foods (Parodi et al., 2018).

Despite several advantages of edible insects, large-scale production for food and feed will lead to novel challenges, including the possible transmission of biological hazards (Eilenberg et al., 2015; van der Fels-Klerx et al., 2018; Garofalo et al., 2019; Osimani and Aquilanti, 2021). Available data suggest that the insect microbiota is complex with marked variations in microbial load and diversity. Insect microbiota is stable and species-specific among the most popular edible insects, such as mealworm larvae and grasshoppers (Garofalo et al., 2019). Also, Tinker and Ottesen (2016) examined the composition and stability of the gut microbiota from the omnivorous cockroach (*Periplaneta americana*). Cockroaches hosted a diverse core gut microbiome that remained stable and resilient to drastic long-term dietary shifts. At the phylum level, the gut microbiota of wild *P. americana* was similar to lab-reared animals. Raw insects generally harbor high numbers of mesophilic aerobes (i.e., *Micrococcus* spp., *Pseudomonas* spp., *Staphylococcus* spp.), bacterial endospores or spore-forming bacteria (*Bacillus* spp., *Clostridium* spp., *Paenibacillus* spp.), *Enterobacteriaceae*, and lactic acid bacteria (*Lactobacillaceae*, *Lactococcus* spp.). Moreover, insects may serve as a host for *Carnobacterium maltaromaticum*. Psychrotrophic aerobes, fungi, and potentially harmful microorganisms (i.e., pathogenic, mycotoxigenic, and spoilage microbes) may also be present based on cultivation-dependent and -independent analyses (Shannon et al., 2001; Hernández-Flores et al., 2015; Stoops et al., 2016; Garofalo et al., 2017; Vandeweyer et al., 2017; Milanović et al., 2018; Wynants et al., 2018).

The presence of distinctive orange pigment-producing colonies during anaerobic cultivation targeted to isolate bifidobacteria from feces of golden lion tamarins (*Leontopithecus rosalia*) and their feed insects led us to further investigate and characterize the isolated strains. Pigment formation has been associated with the pathogenesis of some bacteria. *Serratia marcescens* produces a red pigment prodigiosin, which can contribute to the virulence of the strains, while *Staphylococcus aureus* is an example of a golden pigment staphyloxanthin producer (Liu and Nizet, 2009). According to the study by Liu et al. (2005), the loss of pigmentation translated to a significant decrease in virulence. In contrast, Zhang et al. (2018) observed that virulence of non-pigmented producing *S. aureus* strains was not reduced compared to pigment producers in a murine sepsis model, suggesting that pigment formation is not the only factor causing pathogenesis.

We identified the isolates as members of the *Lactococcus garvieae/petauri/formosensis* group using marker genes and genome comparison. Isolates were characterized biochemically, and the impact of environmental conditions, such as carbon and peptone source and pH, was determined. In this study, we suggest that isolates of *L. garvieae/petauri/formosensis*, which are well-known fish pathogens (Vendrell et al., 2006; Meyburgh et al., 2017; Shahi and Mallik, 2020), produce the orange pigment granadaene, which might add to the pathogenesis of these strains and their potential transmission from feed insect to mammalian gut microbiota and aquacultures.

## Materials and Methods

### Ethics Statement

The sampling of primate feces was performed during routine daily procedures at Olomouc zoo (Czechia). The zoological institution has rigorous standards for animal welfare and is accredited by the European Association of Zoos and Aquaria. All procedures involving animals adhered to recommendations of the “Guide for the Care and Use of Animals” by the Czech University of Life Sciences Prague and to the legal requirements of the Czech Republic for the ethical treatment of non-human primates as well as in accordance with European Directive 2010/63/EU. The research protocol was approved by the Ethic and Animal Care Committee of the Czech University of Life Sciences Prague (protocol number: CZU/17/19).

### Sampling and Cultivation Analysis of Fecal Samples and Feed Insects

In total, 21 fecal samples of a male (*n* = 17), a female (*n* = 3), and their offspring (*n* = 1) golden lion tamarins (*L. rosalia*) were collected to characterize the cultivable anaerobic bacteria and members of the family *Bifidobacteriaceae*. The male animal was in quarantine after transfer from France to Czechia during the sampling period; sampling was performed at Olomouc zoo in 2018–2019. Fecal samples were collected in tubes containing dilution buffer [5 g L^–1^ tryptone, 5 g L^–1^ nutrient broth no. 2, 2.5 g L^–1^ yeast extract (all Oxoid, United Kingdom), 0.5 g L^–1^ L-cysteine, and 1 ml L^–1^ Tween 80 (both Sigma-Aldrich, United States)] and glass pearls for homogenization. Media were prepared in an oxygen−free carbon dioxide environment and then sterilized. Feed insect samples, such as larvae *Tenebrio molitor* and *Z. atratus*, crickets, cockroaches, and grasshoppers, were used for cultivation analysis. Feed insects (10 g) were homogenized and transferred into tubes with dilution buffer (90 ml).

Wilkins–Chalgren Anaerobe agar supplemented with 5 g L^–1^ GMO-Free Soya Peptone (both Oxoid, United Kingdom), 0.5 g L^–1^ L-cysteine, and 1 ml L^–1^ Tween 80 (WSP) was used to determine total counts of anaerobic bacteria. Two selective media WSP-NORF containing mupirocin 100 mg L^–1^, norfloxacin 200 mg L^–1^ (both Oxoid), and 1 ml L^–1^ acetic acid (Sigma-Aldrich) and WSP-MUP (identical composition without norfloxacin) were used for bifidobacterial detection according to the study by Modrackova et al. (2021). Plates were incubated anaerobically using GENbag anaer (bioMérieux, France) at 37°C for 2 days.

### Colony Isolation and Strain Identification

The isolation of putative *Bifidobacterium* spp. from cultivation media (*n* = 102) and consecutive subcultivation was performed using WSP broth under anaerobic conditions at 37°C for 1 day. The fructose-6-phosphate phosphoketolase (F6PPK) test with cetrimonium bromide for cell disruption (Orban and Patterson, 2000) was used to identify members of the *Bifidobacteriaceae* (*n* = 83).

Isolates were identified to the species level using matrix-assisted laser desorption/ionization mass spectrometry (MALDI-TOF MS) with ethanol-formic acid extraction procedure with HCCA matrix solution according to the instructions of the manufacturer (Bruker Daltonik GmbH, Bremen, Germany). Stock cultures of bacteria were stored at −80°C in 30% glycerol. Additional bifidobacterial identification and characterization were not the focus of this article.

Colonies with orange pigmentation (*n* = 16) that were isolated from WSP medium without antibiotics were grown in WSP broth under anaerobic conditions at 37°C for 1 day and were also identified to the species level using MALDI-TOF MS. DNA was isolated using PrepMan Ultra (Applied Biosystems, United States) according to the instructions of the manufacturer and stored at −20°C. Primers fd1 (5′-AGAGTTTGATCCTGGCTCAG-3′) and rP2 (5′-ACGGCTACCTTGTTACGACTT-3′) were used for PCR reaction to amplify 16S rRNA gene (Weisburg et al., 1991). PCR products were purified with E.Z.N.A. Cycle Pure Kit (Omega Bio-Tek, United States) and were Sanger sequenced by Eurofins Genomics (Germany). The obtained sequences were processed in Chromas Lite 2.5.1 (Technelysium Pty Ltd., Australia), BioEdit (Hall, 1999; Hall et al., 2011) with ClustalW algorithm (Thompson et al., 1994), and compared with EZBioCloud^1^ and NCBI^2^ databases.

### Culture Collection Strains and Culture Conditions

*Lactococcus garvieae* B18, which did not produce pigment and had been isolated from the stool of an infant, was obtained from strain collection of the Department of Microbiology, Nutrition and Dietetics, CZU (Czechia). *L. garvieae* subsp. *garvieae* DSM 6783 = ATCC 49156 (originating from kidney of yellowtail), *L. garvieae* DSM 20064 (silage), *L. garvieae* DSM 20385 (raw milk), *L. garvieae* subsp. *garvieae* DSM 20684*^T^* = ATCC 43921*^T^* = NBRC 100934 (bovine mastitis), and *L. petauri* ATCC 159469*^T^* = DSM 104842*^T^* = LMG 30040*^T^* (oral abscess in a sugar glider) were obtained from DSMZ, and granadaene producing *Streptococcus agalactiae* LMG 15088 = ATCC 12386 [no exact information about an origin, group B streptococci (GBS) control strain for media testing] was obtained from LMG. All *Lactococcus* spp. and *S. agalactiae* strains were routinely cultivated in WSP broth. Their purity and identity were checked using phase-contrast microscopy and by MALDI-TOF MS.

### Biochemical, Enzymatic, and Hemolytic Activity Characterization

*Lactococcus* spp. and *S. agalactiae* were initially grown anaerobically at 37°C for 24 h on Wilkins–Chalgren agar without the soya peptone addition. Production of acid connected to pH change, based on the ability to utilize carbohydrates, was determined by using API 50 CH kits (bioMérieux, France) after incubation at 37°C for 48 h. API 20 STREP kits (bioMérieux) were used to determine carbohydrate utilization and enzymatic assay after incubation at 37°C for 4 and 24 h. Enzymatic activity was assessed using API ZYM and Rapid 32 ID A kits (both bioMérieux) after incubation at 37°C for 4–4.5 h. All kits were used according to the instructions of the supplier. The ability of tested strains to induce hemolysis was tested on Tryptone Soya Agar and Columbia agar with 5% of sheep blood (both Oxoid) under aerobic and anaerobic conditions at 37°C for 24 h. All strains were tested at least in two or three biological replicates.

### Impact of Nutrients and pH on Pigment Formation

The growth and cultivation characteristics, such as pigmentation of colonies, were determined at 20, 30, and 37°C under aerobic and anaerobic conditions. Commercial media Wilkins–Chalgren Anaerobe agar, Columbia agar, Brain Heart Infusion agar, Tryptone Soya agar, MRS agar, Rogosa agar (all from Oxoid), Granada agar (bioMérieux), and methotrexate-containing New Granada Medium (De la Rosa et al., 1992) without antibiotic supplementation were tested.

The formation of granadaene pigment was visually evaluated on modified agars containing additional substrates. D-Glucose (in final concentration 1, 2, 5, and 10 g L^–1^), D-mannose (2 or 4 g L^–1^), D-fructose, D-tagatose, D-galactose, D-ribose, D-mannitol, cellobiose, maltose, sucrose, trehalose, gentobiose, *N*-acetylglucosamine, amygdalin, arbutin, esculin, salicin, DL-lactic acid, and D-gluconic acid potassium salt (all in a concentration of 5 g L^–1^; Sigma-Aldrich) were added to the basic Wilkins–Chalgren agar that contains 1 g L^–1^ glucose. The pH of Wilkins–Chalgren agar (pH 6.4 after sterilization) was adjusted by the addition of acetic acid (Sigma-Aldrich) in a concentration to obtain a range from pH 4.1–5.1 (0.5 ml L^–1^ for pH 5.1, 1 ml L^–1^ for pH 4.7, 1.5 ml L^–1^ for pH 4.4, 2 ml L^–1^ for agar pH 4.1). To determine the impact of buffer capacity, 3-(*N*-morpholino)propanesulfonic acid hemisodium salt (11 g L^–1^; MOPS hemisodium salt, Sigma-Aldrich) and disodium hydrogen phosphate (8.5 g L^–1^; Penta, Czechia) were added to Wilkins–Chalgren agar together with glucose (5 g L^–1^). The addition of L-arginine (1 g L^–1^) was tested in combination with basic Wilkins–Chalgren agar containing D-glucose (1 g L^–1^). All used strains were cultivated under anaerobic conditions at 37°C, 24 h.

Moreover, strains were cultivated in basic Wilkins–Chalgren broth supplemented with additional D-glucose for final concentrations 1, 3, 5, or 10 g L^–1^, or D-mannose (2 or 4 g L^–1^). Supernatants were used for pH measurement using Checker Plus-pH HI98100 (Hanna instruments, Czechia) and metabolite analysis.

### Metabolite Analysis Using High-Pressure Liquid Chromatography With Refractive Index Detection

Glucose, mannose, and lactate concentrations present in supernatants were quantified using a 1260 Infinity II (Agilent, Denmark) equipped with a refractive index detector and a Hi-Plex H column (Agilent). H_2_SO_4_ (5 mM) was used as eluent at a flow rate of 0.6 ml min^–1^ at a column temperature of 40°C. Chromatograms were processed with Chromeleon 7.20 (Thermo Scientific). External standards were prepared from 100 and 10 mM solutions (Sigma-Aldrich).

### Bacterial Genome Sequencing and Comparative Genome Analysis

For bacterial genome sequencing, four strains, namely, LG4 and LG26 (originating from feces of golden lion tamarins), I4/6O (*Z. atratus* larvae), and B18 (stool of an infant) were selected. High-molecular-weight DNA was prepared using QIAGEN Genomic Tip/100 G (QIAGEN, Germany) according to the instructions of the manufacturer. SMRTbell template library was prepared according to the instructions from Pacific Biosciences (Menlo Park, CA, United States), following the Procedure & Checklist – Preparing Multiplexed Microbial Libraries Using SMRTbell Express Template Prep Kit 2.0. Briefly, for the preparation of 10 kb libraries 1 μg genomic DNA was sheared using g-tubes (Covaris, MA, United States) according to the instructions of the manufacturer. DNA was end-repaired and ligated to barcoded adapters applying components from the SMRTbell Express Template Prep Kit 2.0 (Pacific Biosciences). Reactions were carried out according to the instructions of the manufacturer. Samples were pooled according to the calculations provided by the Microbial Multiplexing Calculator. Conditions for annealing of sequencing primers and binding of polymerase to purified SMRTbell template were assessed with the Calculator in SMRT link (Pacific Biosciences). Libraries were sequenced on the Sequel*II* (Pacific Biosciences), taking one 15 h movie per SMRT cell.

Long read genome assemblies were performed using the Microbial Assembly protocol included in SMRT link version 8.0.0 applying target genome sizes of 2.1 Mbp (strain B18), 2.4 Mbp (strains I4/6O and LG4), and 2.6 Mbp (strain LG26). All circular chromosomal contigs were adjusted to *dnaA*. Identification of *dnaA* was carried out using BLAST, and circularization and rotation were performed with the genomecirculator.py tool.^3^ Furthermore, up to five circular plasmids (strain I4/6O) were assembled and adjusted to their replication genes.

Strains were taxonomically assigned using the Type Strain Genome Server of the DSM (TYGS, Meier-Kolthoff and Göker, 2019; Meier-Kolthoff et al., 2021). We used EDGAR 2.3 (Blom et al., 2016) for pan-genome analysis and comparison to selected genomes of *S. agalactiae*. A total of 4 genomes were generated in this study (of strains LG4, LG26, I4/6O, and B18), 35 genomes assigned to *L. garvieae*, 2 genomes assigned to *L. petauri* (which were available at NCBI in the fall of 2020), and 10 genomes of *S. agalactiae* (Supplementary Table 1). *L. garvieae* ATCC 49156 (AP009332) was used as a reference genome. A phylogenetic tree based on the core genome using the 41 *Lactococcus* genomes employed in this study was calculated using EDGAR 2.3 (Blom et al., 2016). Briefly, core gene sets were generated using MUSCLE, and the alignments were concatenated to one alignment, which was used as input for tree construction employing the neighbor-joining algorithm as implemented in the PHYLIP package. A similarity matrix was deduced from ANI values (Supplementary Table 2) calculated within EDGAR 2.3 using parameters similar to JSpecies (Richter and Rosselló-Móra, 2009) with an all-against-all comparison. Genes assigned to *cyl* operon of *S. agalactiae* (Spellerberg et al., 2000) were extracted from an EDGAR comparison.

#### Quantitative PCR Analysis to Quantify Total Bacterial Counts

DNA was extracted from fecal and insect samples using the Fast DNA Spin Kit for soil (MP Biomedicals, France), which includes a bead-beating step, according to the instructions of the manufacturer.

The quantitative PCR (qPCR) was performed to determine the abundance of total bacteria for quantitative microbiota profiling (QMP) together with the 16S rRNA gene dataset (described below) using a Roche LightCycle 480 System (Hoffmann-La Roche, Switzerland). Each reaction constituted of 5 μl 2× SensiFAST SYBR No-ROX Mix (Bioline GmbH, Germany), 0.5 μl primer (10 μM), 1 μl of 10-fold diluted DNA, and 3 μl of water. Each sample was analyzed in duplicate. Reaction mixtures were heated to 95°C for 20 s and were subjected to 40 cycles of denaturation at 95°C for 5 s, annealing and extension at 60°C for 60 s, followed by melting curve analysis. In each run, a serial dilution of the corresponding standard was included using a linearized plasmid containing the gene of interest as a template. Correction factors to account for multiple 16S rRNA gene copies (*n* = 5) were used to estimate the cell numbers from gene copies (Stoddard et al., 2015).

#### 16S rRNA Gene Amplicon Sequencing and Analysis

The microbial composition of fecal and intestinal insect microbiota was determined using 16S rRNA gene sequencing. For library preparation, a two-step PCR approach was used targeting the 16S rRNA gene V4 hypervariable region using the primers 515F (5′-GTGCCAGCMGCCGCGGTAA-3′) and 806R (5′-GGACTACHVGGGTWTCTAAT-3′). Sequencing was performed with the Illumina MiSeq platform (Genetic Diversity Centre, ETH Zürich, Switzerland) with 2 × 250 bp read length using v2 chemistry.

16S rRNA gene datasets and libraries generated in previous studies (PRJNA390238, PRJNA418072, PRNJA476046; Supplementary Table 3) were analyzed using the same pipeline. Briefly, Illumina adaptors and gene-specific primers were removed using Atropos (Didion et al., 2017). Sequences were then processed using the DADA2 pipeline, which allows inference of exact amplicon sequence variants (ASVs) (Callahan et al., 2016). Briefly, reads were truncated after 170 and 160 nucleotides for the V4 region and after 260 and 250 nucleotides for the V3V4 region, for forward and reverse reads, respectively. Reads with expected error rates higher than 3 and 4 for the V4 region and 4 and 5 for the V3V4 region, for forward and reverse reads, respectively, were removed. After filtering, error rate learning and ASV inference and read pairs merging were performed on each sequencing run independently (nbase = 2 × 10^8^, minOverlap = 40 nucleotides). ASVs from each run were merged and chimeric sequences were identified and removed using “consensus” method. An additional clustering step was performed using the collapseNoMismatch() function provided by the DADA2 package to confirm that ASVs generated from each run were not artificially identified as distinct. Taxonomy was assigned using GreenGenes 13.8 database (DeSantis et al., 2006) using the functions assign Taxonomy() and add Species() functions provided by the DADA2 package. A total of 914.021 reads was obtained with a mean of 39.143 reads and a median of 38.529 (Supplementary Table 3).

## Results and Discussion

### Bifidobacteria and Other Cultivable Bacteria From Monkey Feces and Their Feed Insects

Fecal samples of a golden lion tamarin family collected during and after transport from France to Czechia, one male, a female, and a baby tamarin located at Olomouc zoo were analyzed for the presence of bifidobacteria. F6PPK-positive isolates belonging to the family *Bifidobacteriaceae* were the dominant bacterial group of cultivable anaerobes with counts of 8.76 ± 0.73 log CFU g^–1^ on WSP-MUP, 7.97 ± 0.98 log CFU g^–1^ on WSP-NORF media compared to total counts on non-selective WSP agar, 9.12 ± 0.60 log CFU g^–1^.

In total, 83 F6PPK-positive isolates were obtained and analyzed by MALDI-TOF MS, 16 isolates were identified to species level as *Bifidobacterium adolescentis* (*n* = 11), *Bifidobacterium catenulatum*/*pseudocatenulatum* (*n* = 4), and *Bifidobacterium pseudolongum* (*n* = 1). The rest of the isolates could not be taxonomically classified on genus and species level due to the limited number of *Bifidobacterium* spp. in the provider’s database and high species variability in primates (Modrackova et al., 2021). In insect feed samples, total anaerobic bacterial (7.62 ± 0.76 log CFU g^–1^) and bifidobacterial counts were lower (WSP-MUP 3.57 ± 1.84 log CFU g^–1^, WSP-NORF 4.32 ± 2.24 log CFU g^–1^). Out of the three F6PPK-positive isolates (*n* = 3), one isolate from *Z. atratus* larvae was identified as *Bifidobacterium psychraerophilum*.

**FIGURE 1:**
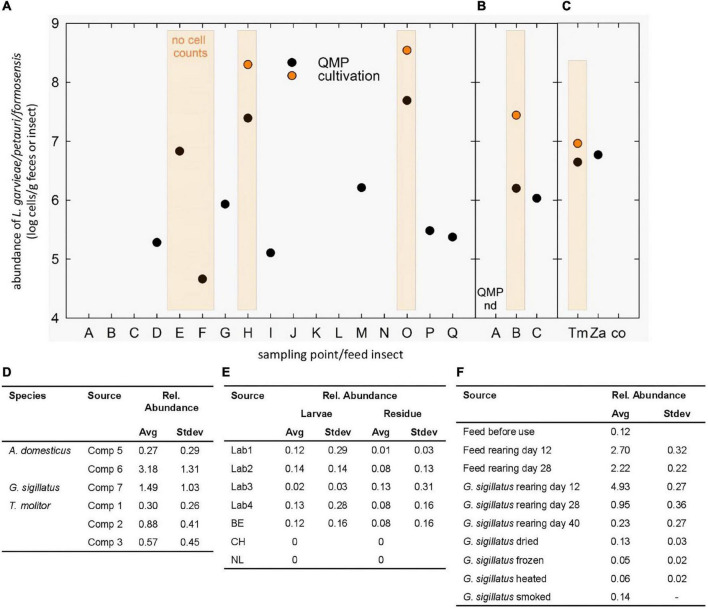
Occurrence of *L. garvieae/petauri/formosensis* in monkeys and feed insects. **(A-C)** Cell counts of *L. garvieae/petauri/formosensis* in male **(A)** and female **(B)** monkeys and feed insects **(C)** determined by QMP and cultivation at different sampling points. The minimal detection limit of QMP was around log 4 cells g^–1^. Orange-shaded columns indicate the presence of orange-pigmented colonies on WSP agar. No cell count, orange-pigmented colonies were detected, but cell counts were not recorded. **(D-F)** Reanalysis of available 16S rRNA gene datasets to determine the occurrence of *L. garvieae/petauri/formosensis* in two crickets and one mealworm species reared at different companies (**D**; Vandeweyer et al., 2017), of mealworms and their residues reared in different labs and countries (**E**; Vandeweyer et al., 2018), and of the cricket *G. sigillatus* during rearing and after processing (**F**; Wynants et al., 2018).

Even though the medium for the total bacterial numbers was intended as a control medium, our interest was attracted by the orange colonies detected in five fecal samples of golden lion tamarin at counts around 8 log CFU g^–1^ (Figure 1) and 7 log CFU g^–1^ of *Z. atratus* larvae. Orange pigmented colonies were identified by MALDI-TOF MS as *L. garvieae/petauri*. *Lactococcus* spp. are important cultures in the dairy industry, various sub/species are able to colonize other various plant and animal environments (Kelleher et al., 2017). *L. garvieae* has been isolated from aquatic and terrestrial animals and from various food products, such as milk, meat, fish, vegetables, cereals, and feedstuffs (Gibello et al., 2016). This wide distribution is likely related to the ability of *L. garvieae* to adapt and survive in various environmental conditions (Ferrario et al., 2013; Gibello et al., 2016). The taxonomically very closed related *L. petauri* also produced a bright orange pigmented phenotype (Goodman et al., 2017).

### Identity of Pigment-Producing Lactococci Isolated From Feed Insects and Golden Lion Tamarins

Next, we further taxonomically assigned the isolates (*n* = 16) from monkey feces and insects with orange pigment production. Based on the sequence of the 16S rRNA gene, strains had similarities >99.15% to strains of *L. petauri*, *L. garvieae*, and *L. formosensis*, whose 16S rRNA gene sequences differed only in a few nucleotides. These observations indicate that 16S rRNA gene-based analysis did not allow reliable differentiation of lactococci on the species level, which was in accordance with MALDI-TOF MS identification. To enable additional taxonomic and functional characterization, three strains, LG4 and LG26, originating from feces of golden lion tamarins, and I4/6O from *Z. atratus* larvae, were selected for whole-genome sequencing, further genotyping and phenotyping characterization, and were compared to strain B18, which had been isolated from the stool of an infant, and to the public collection and type strains.

### Persistence and Occurrence of *Lactococcus garvieae/petauri/formosensis* in Monkey Fecal and Insect Gut Microbiomes

As *L. garvieae/petauri/formosensis* were recovered from monkey feces and feed insects, we monitored their abundance in feces of the two adult animals by 16S rRNA gene sequencing and cultivation. Orange colonies (around 8 log cells g^–1^) were obtained from the male and female animals at five time points (Figure 1), while the relative abundance of ASVs assigned to *Lactococcus* (*Lactococcus* sp. ASV089, and *L. garvieae* ASV090 and ASV091) ranged from 0 to 1.9%, indicating that lactococci were recurrently detectable at fluctuating relative abundance levels. The absolute abundance in samples with detectable levels ranged from 5.3 to 7.7 log cells g^–1^ feces (Figure 1). Cell counts of *L. garvieae/petauri/formosensis* in feed larvae *T. molitor* and *Z. atratus* were 6.2 and 7.0 log cells g^–1^ constituting around 18.5 and 12% of the total microbiota. In cockroaches, *L. garvieae/petauri/formosensis* represented 0.02% of the microbiota (3.8 log g^–1^).

To compare the occurrence of *L. garvieae/petauri/formosensis* in feed insects to other studies, we additionally reanalyzed available 16S rRNA gene libraries using the same analytical pipeline. *L. garvieae/petauri/formosensis* were recovered from two crickets *Gryllodes sigillatus* and *Acheta domesticus*, and from the mealworm *T. molitor* at 0.3–3.2% relative abundance (reanalyzed from Vandeweyer et al., 2017). In *G. sigillatus* and the corresponding cricket feed, *L. garvieae/petauri/formosensis* were detected during rearing (Figure 1) with the relative abundance being reduced after drying, freezing, heating, and smoking (reanalyzed from Vandeweyer et al., 2018). Moreover, *L. garvieae/petauri/formosensis* were recovered from laboratory and industrially reared *Hermetia* and the residues (Figure 1; reanalyzed from Wynants et al., 2018).

Our results, together with other studies (Garofalo et al., 2017; Smith et al., 2017), strongly emphasize the colonization of feed insects with *L. garvieae/petauri/formosensis*, which might lead to a transfer to mammals. In agreement, *L. petauri* was isolated from an insectivorous sugar glider (Goodman et al., 2017).

### Pigment-Producing Strains Are Hemolytic

**FIGURE 2:**
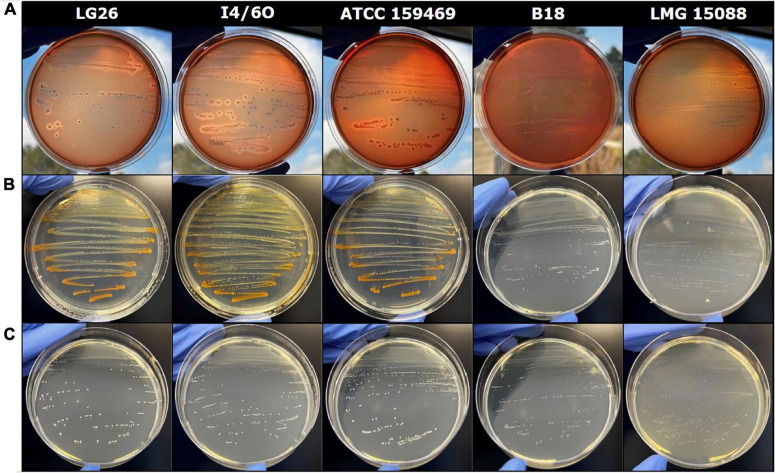
Effect of variable conditions on orange pigment formation. **(A-C)** Induction of hemolysis on Columbia agar with 5% of sheep blood **(A)**, orange pigment formation on Wilkins–Chalgren agar **(B)**, or Wilkins–Chalgren agar at levels with ≥3 g L^–1^ of D-glucose **(C)**, all plates were cultivated under anaerobic conditions at 37°C; *L. garvieae/petauri/formosensis* (LG26, golden lion tamarin origin; I4/6O, insect origin; 159469*^T^*, oral abscess in a sugar glider; B18, infant origin), *S. agalactiae* (LMG 15088, unknown origin).

An orange pigment contributes to pathogenesis of GBS (Liu and Nizet, 2009). This pigment is a cytotoxic lipid known as granadaene (Armistead et al., 2020a) and is composed of an ornithine rhamnolipid that contains a linear chain of 12 conjugated double bonds that are connected to the amino acid ornithine at one end and the sugar rhamnose at the other (Rosa-Fraile et al., 2006; Paradas et al., 2012). This pigment is considered as the hemolysin of *S. agalactiae* that likely contributes to virulence due to its broad-spectrum activity (Spellerberg et al., 2000; Nizet, 2002; Rosa-Fraile et al., 2014). Only granadaene-producing *S. agalactiae* LMG 15088 and the pigment-producing strains ATCC 159469*^T^*, LG4, LG26, and I4/6O showed β-hemolytic activity (Figure 2A), providing strong indication that selected strains of *L. garvieae* produce a similar granadaene like pigment.

*Lactococcus garvieae* has been linked to human and animal diseases. The first documented occurrence of *L. garvieae* has been in ruminants with subclinical mastitis (Garvie et al., 1981). *L. garvieae* is also considered a fish pathogen (Vendrell et al., 2006; Meyburgh et al., 2017; Shahi and Mallik, 2020) and has been associated with endocarditis of humans, bacteremia, and pneumonia of pigs. Human *L. garvieae* infections have been related to the consumption or the handling of contaminated raw fish or seafood (Gibello et al., 2016), yet, whether pigment-producing strains contributed to the reported pathogenesis has not been investigated.

### Comparative Genomics of *Lactococcus garvieae/petauri/formosensis* Group Strains

In *S. agalactiae*, the formation of granadaene and another GBS virulence factor, the pore-forming β-hemolysin/cytolysin, has been linked to the presence of the *cyl* operon (Spellerberg et al., 2000). Expression of the genes of the *cyl* operon is sufficient for granadaene production in heterologous bacterial hosts (Armistead et al., 2020b). We generated genomes of isolates LG4, LG26, I4/6O, and B18 to determine whether a similar operon would be present in pigment-producing *Lactococcus* spp. Strains had an average genome size of 2.41 ± 0.27 Mbp (Supplementary Table 1). The average GC content was 37.75 ± 0.44%. We used EDGAR, to identify and analyze the pan-genome, including all genomes publicly available in the fall of 2020.

The total number of coding sequences (CDS) ranged from 1959 to 2581, while the core genome encompassed 1106 CDS (16.1%). Based on TYGS analysis, I4/6O was identified as *L. formosensis*, while LG4, LG26, and B18 as *L. petauri*.

**FIGURE 3:**
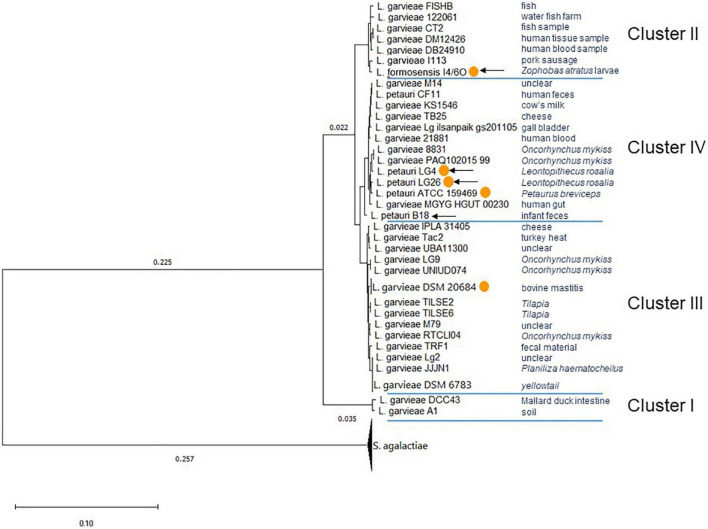
Phylogenetic tree of *L. garvieae/petauri/formosensis* by core genomes. The phylogenetic tree was built using EDGAR and the neighbor-joining algorithm as implemented in the PHYLIP package with rerooting. The tree is based on the core genome of 50 genomes, comprising 478 core genes each, 23,900 in total. The phylogenetic tree was built from 178,713 AA-residues per genome, 8,935,650 in total. Multiple genome copies of one strain were compressed. Origin of strains was retrieved from NCBI’s genome server. With orange circles: strains that have confirmed pigment formation. With arrows: strains whose genomes were generated in this study.

Both the phylogenetic tree (Figure 3) and ANI analysis (Supplementary Table 2), using *S. agalactiae* strains as an outgroup, identified four clusters with the *L. garvieae*, *L. petauri*, and *L. formosensis* isolates. The ANI between the different clusters was below 95%. Taken together, these data support the existence of three species within the *L. garvieae/petauri/formosensis* group; cluster II was represented by our isolate *L. formosensis* I4/6O, cluster III by *L. garvieae* isolates, including type strain DSM 20684*^T^*, and cluster IV by our isolates *L. petauri* ATCC 159469*^T^*, LG4, LG26, and B18 plus other *L. garvieae* isolates (Figure 3). There was no clear relationship between cluster assignment and origin of the individual strains.

**FIGURE 4:**
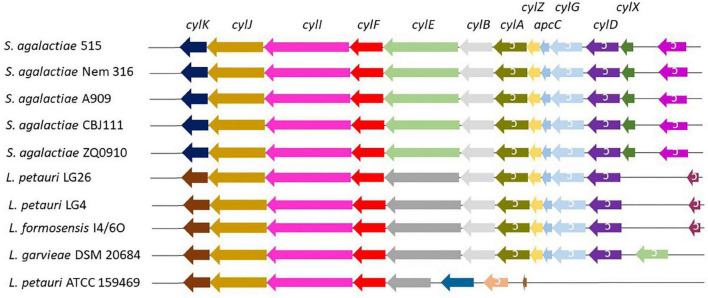
*Cyl* operon in *S. agalactiae* and *L. garvieae/petauri/formosensis*. Analogs of the *cyl* operon of three pigment-producing *S. agalactiae* (Six et al., 2016, see below) in *L. garvieae/petauri/formosensis* were extracted using the genome browser function implemented in Edgar. Genes are not drawn according to scale.

A total of 51 genomes (Supplementary Table 1) were screened for the presence of *cyl* operons (Figure 4). *Cyl* operons were detected in LG4, LG26 (both cluster IV), and I4/6O (cluster II), but were not detected in B18 (cluster IV) (Figure 3). A *cyl* operon was present in genomes of the type strains *L. petauri* ATCC 159469 (cluster IV) and *L. garvieae* DSM 20684 (cluster III). These data indicate that few strains of *L. garvieae/petauri/formosensis* group can form granadaene; the ability to form granadaene did not relate to the phylogenetic relationship. The *cyl* operon, which is made up of 12 genes (*cylX*, *cylD*, *cylG*, *acpC*, *cylZ*, *cylA*, *cylB*, *cylE*, *cylF*, *cylI*, *cylJ*, and *cylK*), is unique to GBS (Rosa-Fraile et al., 2014). However, strains of *L. garvieae/petauri/formosensis* group lacked some of these genes (Figure 4).

### Impact of Carbohydrate Source and pH on Granadaene Production

To further investigate granadaene production of lactococci, we compared biochemical profiles and the impact of environmental conditions on nine *Lactococcus* and one *Streptococcus* strain. Fermentation and enzymatic profiles of golden lion tamarin and insect isolates were similar to *L. petauri* ATCC 159469*^T^*. Strains produced acid from D-ribose, D-galactose, D-glucose, D-fructose, D-mannose, *N*-acetylglucosamine, amygdalin, arbutin, esculin, salicin, D-cellobiose, D-maltose, sucrose, D-trehalose, gentobiose, and D-tagatose (Supplementary Table 4). The utilization of D-mannitol by the strain ATCC 159469*^T^* was weak compared to LG4, LG26, and I4/6O. The insect strain I4/6O did not produce acid from sucrose and D-tagatose.

Strains LG4, LG26, I4/6O, and ATCC 159469*^T^* were able to produce orange pigment on Wilkins–Chalgren (Figure 2B), Columbia, Brain Heart Infusion, and Tryptone Soya agar at 20, 30, and 37°C, when incubated at aerobic and anaerobic conditions but not on MRS or Rogosa agar. In contrast, *L. garvieae* DSM 6783, DSM 20064, DSM 20385, DSM 20684*^T^*, strain B18, and *S. agalactiae* LMG 15088 grew but did not produce pigment *in vitro*. Granadaene-positive lactococci produced some orange pigment on Granada medium without antibiotics (De la Rosa et al., 1992), and not on commercial Granada agar. In contrast, *S. agalactiae* LMG 15088 produced clear orange pigment on both Granada agars.

These observations led us to compare media compositions to identify compounds impacting pigment formation. D-Glucose addition at levels ≥3 g L^–1^ or D-mannose addition (≥2 g L^–1^) to Wilkins–Chalgren medium inhibited pigment formation (Figure 2C) while reducing the pH in the supernatant to a value of about 4 (Supplementary Table 5). Similarly, fermentation of D-fructose, D-mannose, D-cellobiose, and D-trehalose (concentration of 5 g L^–1^ in Wilkins–Chalgren agar) suppressed pigment formation compared to other tested fermentable substrates (Table 1).

**TABLE 1:** Effect of different carbohydrates in Wilkins–Chalgren medium on the orange pigment formation.

Effect of substrate (5 g L^–1^) in Wilkins–Chalgren on granadaene production	Orange pigment production (positive/negative)									
	ATCC	ČZU	ČZU	ČZU	DSM	DSM	DSM	DSM	ČZU	LMG
	159469*^T, G^*	LG4*^G^*	LG26*^G^*	I4/6O*^G^*	20064	6783	20684*^T, G^*	20385	B18	15088*^G^*
D-Ribose	+	+	+	+	−	−	−	−	−	−
D-Galactose	+	+	+	+	−	−	−	−	−	−
D-Glucose	−	−	−	−	−	−	−	−	−	−
D-Fructose	−	−	−	−	−	−	−	−	−	−
D-Mannose	−	−	−	−	−	−	−	−	−	−
D-Mannitol	+	+	+	+	−	−	−	−	−	−
*N*-Acetylglucosamine	+	+	+	+	−	−	−	−	−	−
Amygdalin	+	+	+	+	−	−	−	−	−	−
Arbutin	+	+	+	+	−	−	−	−	−	−
Esculin ferric citrate	+	+	+	+	−	−	−	−	−	−
Salicin	+	+	+	+	−	−	−	−	−	−
Cellobiose	−	−	−	−	−	−	−	−	−	−
Maltose	+	+	+	+	−	−	−	−	−	−
Sucrose	+	+	+	+	−	−	−	−	−	−
Trehalose	−	−	−	−	−	−	−	−	−	−
Gentiobiose	+	+	+	+	−	−	−	−	−	−
Tagatose	+	+	+	+	−	−	−	−	−	−
Gluconic acid potassium salt	+	+	+	+	−	−	−	−	−	−
Lactose	+	+	+	+	−	−	−	−	−	−

*^T^Type strain, ^G^strain with cyl operon and prediction to produce granadaene pigment, (+) positive, and (−) negative.*

To further test the impact of pH, we added acetic acid to the Wilkins–Chalgren medium. Pigmentation was visible at pH 5.1 and 6.4 but not at pH 4.7 or less. The addition of the buffering compound MOPS (p*K*a 7.20) to the Wilkins–Chalgren medium restored pigment formation. The addition of L-arginine (≥1 g L^–1^) into Wilkins–Chalgren media containing glucose (≥3 g L^–1^) that commonly suppressed granadaene production also recovered pigment formation. Pigment-producing lactococci and *S. agalactiae* LMG 15088 were positive for arginine hydrolase, arginine dihydrolase, arginine iminidase, arylamidase, and glutamic acid decarboxylase activity (Supplementary Table 4). The release of alkaline molecules, such as ammonia, from arginine contributes to the acid resistance of lactic acid bacteria (Shabayek and Spellerberg, 2017). Likewise, amino acid decarboxylation is generally considered as among the biochemical systems allowing lactic acid bacteria to counteract acidic stress and obtaining metabolic energy (Laroute et al., 2016). Taken together, data strongly indicate the pH dependency of granadaene pigment formation in lactococci.

### Impact of Peptide Source on Granadaene Production

For the formation of red-brick colonies of GBS in Granada medium, the presence of the peptide Ile-Ala-Arg-Arg-His-Pro-Tyr-Phe in the culture medium is essential. This peptide is produced only during the hydrolysis with pepsin of mammal albumin (Rosa-Fraile et al., 1999) and is a component of protease peptone (N3, Difco). In contrast, *Lactococcus* spp. isolated from monkeys (LG4, LG26) and feed insects (I4/6O), and *L. petauri* ATCC 159469*^T^* were able to produce the orange pigment on low sugar media in the presence of other types of peptones; gelatin peptone L92 (Wilkins–Chalgren agar), protease peptone L85 (Brain Heart Infusion agar), special peptone L72 (Columbia agar), or pancreatic digest of casein and enzymatic digest of soya bean (Tryptone Soya agar). Therefore, the production of granadaene by *Lactococcus* spp. is probably not connected to mammalian albumin (Rosa-Fraile et al., 1999), which might indicate adaptation to different hosts.

Interestingly, we detected also differences between the lactococci strains with *cyl* operon in the pigment production under the tested conditions. The type strains of *L. garvieae* DSM 20684 and *L. petauri* ATCC 159469 harbored the *cyl* operon, but with alterations in gene organization compared to isolates *L. petauri* (LG4 and LG26) and *L. formosensis* I4/6O (Figure 4). The orange pigment phenotype was observed by strains isolated from golden lion tamarin and insect origins, and *L. petauri* ATCC 159469*^T^*, but not by *L. garvieae* DSM 20684*^T^*.

## Conclusion

In this study, we showed that *L. garvieae/petauri/formosensis* group encompasses at least four clusters. Strains from different clusters formed the orange pigment granadaene similar to *S. agalactiae*. Feed insects were identified as a regular source of *L. garvieae/petauri/formosensis* with a possible transfer to the gut microbiota of humans and animals, and also for aquacultures, where insects and their products are used for feeding. This study can provide further guidance on the identification and evaluation of risk factors, such as granadaene, that are associated with regular insect consumption to guarantee the safety of insects as food and feed sources in future diets.

## Data Availability Statement

The datasets presented in this study can be found in online repositories. The names of the repository/repositories and accession number(s) can be found in the article/Supplementary Material.

## Author Contributions

VN-B and ClS designed the study. VN-B, MM, MS, CaS, and ClS performed the experiments. VN-B, ARG, BB, JB, and ClS analyzed the data. VN-B, NM, and ClS prepared the tables and figures and wrote the manuscript. All authors edited the manuscript and contributed to the article and approved the submitted version.

## Conflict of Interest

CaS and BB were employed by the German Collection of Microorganisms and Cell Cultures GmbH. The remaining authors declare that the research was conducted in the absence of any commercial or financial relationships that could be construed as a potential conflict of interest.

## Publisher’s Note

All claims expressed in this article are solely those of the authors and do not necessarily represent those of their affiliated organizations, or those of the publisher, the editors and the reviewers. Any product that may be evaluated in this article, or claim that may be made by its manufacturer, is not guaranteed or endorsed by the publisher.

## Footnotes

1
https://www.ezbiocloud.net/

2
https://www.ncbi.nlm.nih.gov/

3
https://github.com/boykebunk/genomefinish
