# Supplementary material for: Feed Insects as a Reservoir of Granadaene-Producing Lactococci
Source: Front Microbiol. 2022 May 9;13:848490. doi: 10.3389/fmicb.2022.848490 (PMC9125021; doi:10.3389/fmicb.2022.848490)
Supplement: Supplementary file 3 [file Table_3.DOCX]

**Table S3.** Analysed 16S rRNA gene datasets and libraries generated in previous studies and the current dataset

| Study ID | Reference | Number of Samples | Total Observed Species | Statistics of reads per sample | | | | | |
| --- | --- | --- | --- | --- | --- | --- | --- | --- | --- |
|  |  |  |  | Min | Max | Median | Average | SD | Total Reads |
| PRJNA390238 | ([Vandeweyer et al., 2017](#_ENREF_43)) | 34 | 1 351 | 58 127 | 106 860 | 80 882 | 79 818 | 11 308 | 2 713 819 |
| PRJNA418072 | ([Vandeweyer et al., 2018](#_ENREF_44)) | 40 | 1 964 | 45 290 | 118 888 | 92 558 | 91 310 | 16 561 | 3 652 387 |
| PRNJA476046 | ([Wynants et al., 2018](#_ENREF_48)) | 110 | 4 937 | 13 422 | 936 812 | 48 545 | 67 627 | 108 830 | 7 438 974 |
| PRJEB48009 | This Study | 24 | 850 | 18 366 | 60 279 | 38 520 | 39 143 | 9 944 | 914 201 |

**References:**

Vandeweyer, D., Crauwels, S., Lievens, B., and Van Campenhout, L. (2017). Microbial counts of mealworm larvae (Tenebrio molitor) and crickets (Acheta domesticus and Gryllodes sigillatus) from different rearing companies and different production batches. *International Journal of Food Microbiology* 242**,** 13-18. doi: 10.1016/j.ijfoodmicro.2016.11.007.

Vandeweyer, D., Wynants, E., Crauwels, S., Verreth, C., Viaene, N., Claes, J., et al. (2018). Microbial dynamics during industrial rearing, processing, and storage of tropical house crickets (Gryllodes sigillatus) for human consumption. *Applied and Environmental Microbiology* 84(12). doi: 10.1128/AEM.00255-18.

Wynants, E., Crauwels, S., Verreth, C., Gianotten, N., Lievens, B., Claes, J., et al. (2018). Microbial dynamics during production of lesser mealworms (Alphitobius diaperinus) for human consumption at industrial scale. *Food Microbiology* 70**,** 181-191. doi: 10.1016/j.fm.2017.09.012.
