# Supplementary material for: Feed Insects as a Reservoir of Granadaene-Producing Lactococci
Source: Front Microbiol. 2022 May 9;13:848490. doi: 10.3389/fmicb.2022.848490 (PMC9125021; doi:10.3389/fmicb.2022.848490)
Supplement: Supplementary file 4 [file Table_4.DOCX]

**Table S4.** (A, B, C, D) Biochemical characterization of selected strains using commercial kits (Biomérieux); API 50 CH (A), Rapid 32 ID A (B), API 20 STREP (C), API ZYM (D)

| **(A) API 50 CH** | ATCC | ČZU | ČZU | ČZU | DSM | DSM | DSM | DSM | ČZU | LMG |
| --- | --- | --- | --- | --- | --- | --- | --- | --- | --- | --- |
| Active ingredients: | **159469^T, G^** | **LG4^G^** | **LG26^G^** | **I4/6O^G^** | **20064** | **6783** | **20684^T, G^** | **20385** | **B18** | **15088^G^** |
| Control | - | - | - | - | - | - | - | - | - | - |
| Glycerol | - | - | - | - | - | - | - | - | - | - |
| Erythritol | - | - | - | - | - | - | - | - | - | - |
| D-arabinose | - | - | - | - | - | - | - | - | - | - |
| L-arabinose | - | - | - | - | - | - | - | - | - | - |
| D-ribose | + | + | + | + | + | + | + | + | + | + |
| D-xylose | - | - | - | - | - | - | - | - | - | - |
| L-xylose | - | - | - | - | - | - | - | - | - | - |
| D-xylose | - | - | - | - | - | - | - | - | - | - |
| Methyl-βD-xylopyranoside | - | - | - | - | - | - | - | - | - | - |
| D-galactose | + | + | + | + | + | + | + | + | + | + |
| D-glucose | + | + | + | + | + | + | + | + | + | + |
| D-fructose | + | + | + | + | + | + | + | + | + | + |
| D-mannose | + | + | + | + | + | + | + | + | + | + |
| L-sorbose | - | - | - | - | - | - | - | - | - | - |
| L-rhamnose | - | - | - | - | - | - | - | - | - | - |
| Dulcitol | - | - | - | - | - | - | - | - | - | - |
| Inositol | - | - | - | - | - | - | - | - | - | - |
| D-mannitol | -/+ | + | + | + | - | + | + | + | + | - |
| D-sorbitol | - | - | - | - | - | - | - | - | - | - |
| Methyl-αD-mannopyranoside | - | - | - | - | - | - | - | - | - | - |
| Methyl-αD-glucopyranoside | - | - | - | - | - | - | - | - | - | - |
| N-acetylglucosamine | + | + | + | + | + | + | + | + | + | + |
| Amygdalin | + | + | + | + | + | + | + | + | + | - |
| Arbutin | + | + | + | + | + | + | + | + | + | -/+ |
| Esculin ferric citrate | + | + | + | + | + | + | + | + | + | - |
| Salicin | + | + | + | + | + | + | + | + | + | + |
| D-cellobiose | + | + | + | + | + | + | + | + | + | - |
| D-maltose | + | + | + | + | + | + | + | + | + | + |
| D-lactose (bovine origin) | - | - | - | - | - | - | - | - | - | - |
| D-melibiose | - | - | - | - | - | - | - | - | - | - |
| D-saccharose (sucrose) | + | + | + | - | - | - | - | + | + | + |
| D-trehalose | + | + | + | + | + | + | + | + | + | + |
| Inulin | - | - | - | - | - | - | - | - | - | - |
| D-melezitose | - | - | - | - | - | - | - | - | - | - |
| D-raffinose | - | - | - | - | - | - | - | - | - | - |
| Amidon (starch) | - | - | - | - | - | - | - | - | - | - |
| Glycogen | - | - | - | - | - | - | - | - | - | - |
| Xylitol | - | - | - | - | - | - | - | - | - | - |
| Gentiobiose | + | + | + | + | + | + | + | + | + | - |
| D-turanose | - | - | - | - | - | - | - | - | - | - |
| D-lyxose | - | - | - | - | - | - | - | - | - | - |
| D-tagatose | + | + | + | - | - | - | - | + | + | - |
| D-fucose | - | - | - | - | - | - | - | - | - | - |
| L-fucose | - | - | - | - | - | - | - | - | - | - |
| D-arabitol | - | - | - | - | - | - | - | - | - | - |
| L-arabitol | - | - | - | - | - | - | - | - | - | - |
| Potassium gluconate | -/+ | -/+ | -/+ | -/+ | -/+ | -/+ | -/+ | -/+ | -/+ | - |
| Potassium-2-ketogluconate | - | - | - | - | - | - | - | - | - | - |
| Potassium-5-ketogluconate | - | - | - | - | - | - | - | - | - | - |
|  |  |  |  |  |  |  |  |  |  |  |
| **(B) Rapid 32 ID A** | ATCC | ČZU | ČZU | ČZU | DSM | DSM | DSM | DSM | ČZU | LMG |
| Reactions/ Enzymes: | **159469^T, G^** | **LG4^G^** | **LG26^G^** | **I4/6O^G^** | **20064** | **6783** | **20684^T, G^** | **20385** | **B18** | **15088^G^** |
| Urease | - | - | - | - | - | - | - | - | - | - |
| Arginine hydrolase | + | + | + | + | + | + | + | + | + | + |
| α-galactosidase |  | - | - | - | - | - | - | - | - | - |
| ß-galactosidase | - | - | - | - | - | - | - | - | - | - |
| ß-galactosidase 6 phosphate | - | - | - | - | - | - | - | - | - | - |
| α-glucosidase | - | - | - | - | - | - | - | - | - | + |
| ß-glucosidase | + | + | + | + | + | + | + | + | + | - |
| α-arabinosidase | - | - | - | - | - | - | - | - | - | - |
| ß-glucuronidase | - | - | - | - | - | - | - | - | - | - |
| N-acetyl-ß-glucosaminidase | - | -/+ | -/+ | -/+ | -/+ | - | - | - | - | - |
| Mannose fermentation | + | + | + | + | + | + | + | + | + | + |
| Raffinose fermentation | - | - | - | - | - | - | - | - | - | - |
| Glutamic acid decarboxylase | + | + | + | + | - | - | - | - | - | + |
| α-fucosidase | - | - | - | - | - | - | - | - | - | - |
| Nitrates reduction | - | - | - | - | - | - | - | - | - | - |
| Indole formation | - | - | - | - | - | - | - | - | - | - |
| Alcalic phosphatase | - | - | - | - | - | - | - | - | - | + |
| Arginine arylamidase | + | + | + | + | + | + | + | + | + | + |
| Proline arylamidase | - | - | - | - | - | - | - | - | - | - |
| Leucyl glycine arylamidase | + | + | + | + | -/+ | + | + | + | -/+ | - |
| Phenylalanine arylamidase | + | + | + | + | + | + | + | + | + | + |
| Leucine arylamidase | + | + | + | + | + | + | + | + | + | + |
| Pyroglutamic acids arylamidase | -/+ | + | + | + | -/+ | -/+ | -/+ | -/+ | -/+ | - |
| Tyrosine arylamidase | + | + | + | + | + | + | + | + | + | + |
| Alanine arylamidase | + | + | + | + | + | + | + | + | + | + |
| Glycine arylamidase | + | + | + | + | + | + | + | + | + | + |
| Histidine arylamidase | + | + | + | + | + | + | + | + | + | + |
| Glutamyl glutamic acid arylamidase | - | - | - | - | - | - | - | - | - | - |
| Serin arylamidase | + | + | + | + | + | + | + | + | + | + |
|  |  |  |  |  |  |  |  |  |  |  |
|  |  |  |  |  |  |  |  |  |  |  |
| **(C) API 20 STREP** | ATCC | ČZU | ČZU | ČZU | DSM | DSM | DSM | DSM | ČZU | LMG |
| Reactions/ Enzymes: | **159469^T, G^** | **LG4^G^** | **LG26^G^** | **I4/6O^G^** | **20064** | **6783** | **20684^T, G^** | **20385** | **B18** | **15088^G^** |
| Acetonin production (Voges–Proskauer test) | + | + | + | + | + | + | + | + | + | + |
| Hydrolysis (hippuric acid) | -/+ | -/+ | -/+ | -/+ | - | - | - | - | - | -/+ |
| ß-Glucosidase hydrolysis (esculin) | + | + | + | + | + | + | + | + | + | -/+ |
| Pyrrolidonyl arylamidase | - | - | - | - | - | - | - | - | - | - |
| α-Galactosidase | - | - |  | - | - | - | - | - | - | - |
| ß-Glucuronidase | - | - | - | - | - | - | - | - | - | - |
| ß-Galactosidase | - | - | - | - | - | - | - | - | - | - |
| Alkaline phosphatase | - | - | - | - | - | - | - | - | - | + |
| Leucine aminopeptidase | + | + | + | + | + | + | + | + | + | + |
| Arginine dihydrolase | + | + | + | + | -/+ | -/+ | + | + | + | -/+ |
| Active ingredients: |  |  |  |  |  |  |  |  |  |  |
| D-Ribose | + | + | + | + | -/+ | + | + | + | + | + |
| L-Arabinose | - | - | - | - | - | - | - | - | - | - |
| D-Mannitol | - | + | + | + | - | - | + | + | + | + |
| D-Sorbitol | - | - | - | - | - | - | - | - | - | - |
| D-Lactose (bovine origin) | - | - | - | - | - | - | - | - | - | - |
| D-Trehalose | + | + | + | + | + | + | + | + | + | + |
| Inulin | - | - | - | - | - | - | - | - | - | - |
| D-Raffinose | - | - | - | - | - | - | - | - | - | - |
| Starch | - | -/+ | -/+ | -/+ | - | - | -/+ | -/+ | -/+ | -/+ |
|  |  |  |  |  |  |  |  |  |  |  |
| **(D) API ZYM** | ATCC | ČZU | ČZU | ČZU | DSM | DSM | DSM | DSM | ČZU | LMG |
| Enzyme assayed for: | **159469^T, G^** | **LG4^G^** | **LG26^G^** | **I4/6O^G^** | **20064** | **6783** | **20684^T, G^** | **20385** | **B18** | **15088^G^** |
| Control | - | - | - | - | - | - | - | - | - | - |
| Alkaline phosphatase | - | - | - | - | - | - | - | - | - | + |
| Esterase (C 4) | - | - | - | - | - | - | - | - | - | - |
| Esterase Lipase (C 8) | - | - | - | - | - | - | - | - | - | - |
| Lipase (C 14) | - | - | - | - | - | - | - | - | - | - |
| Leucine arylamidase | + | + | + | + | + | + | + | + | + | + |
| Valine arylamidase | - | - | - | - | - | - | - | - | - | - |
| Cystine arylamidase | - | - | - | - | - | - | - | - | - | - |
| Trypsin | - | - | - | - | - | - | - | - | - | - |
| α-chymotrypsin | + | -/+ | -/+ | -/+ | -/+ | -/+ | -/+ | -/+ | -/+ | - |
| Acid phosphatase | + | + | + | + | -/+ | + | -/+ | + | + | + |
| Naphthol-AS-BI-phosphohydrolase | + | + | + | + | -/+ | -/+ | + | + | -/+ | + |
| α-galactosidase | - | - | - | - | - | - | - | - | - | - |
| β-galactosidase | - | - | - | - | - | - | - | - | - | - |
| β-glucoronidase | - | - | - | - | - | - | - | - | - | - |
| α-glucosidase | - | - | - | - | - | - | - | - | - | + |
| β-glucosidase | + | + | + | + | -/+ | + | + | + | -/+ | - |
| N-acetyl-β-glucosaminidase | - | - | - | - | - | - | - | - | - | - |
| α-mannosidase | - | - | - | - | - | - | - | - | - | - |
| α-fucosidase | - | - | - | - | - | - | - | - | - | - |

**Footnotes:** ^T^ type strain, ^G^ strain with *cyl* operon and prediction to produce granadaene pigment; (+) positive, (-) negative, (-/+) week reaction
