# Supplementary material for: Feed Insects as a Reservoir of Granadaene-Producing Lactococci
Source: Front Microbiol. 2022 May 9;13:848490. doi: 10.3389/fmicb.2022.848490 (PMC9125021; doi:10.3389/fmicb.2022.848490)
Supplement: Supplementary file 5 [file Table_5.DOCX]

**Table S5.** Effect of carbohydrate addition on pH and lactate formation. pH and lactate concentrations indicate the mean of two biological replicates.

| **Effect of substrate in Wilkins-Chalgren broth on pH of grown culture** | pH and lactate levels (**pH**/mM) | | | | | | | | | | |
| --- | --- | --- | --- | --- | --- | --- | --- | --- | --- | --- | --- |
|  | ATCC | ČZU | ČZU | ČZU | DSM | DSM | DSM | DSM | ČZU | LMG |  |
|  | **159469^T, G^** | **LG4^G^** | **LG26^G^** | **I4/6O^G^** | **20064** | **6783** | **20684^T, G^** | **20385** | **B18** | **15088^G^** |  |
| D-glucose (1 g L^-1^) | **5.28**/nt | **5.41**/21.33 | **5.41**/22.16 | **5.01**/20.35 | **5.12**/20.58 | **5.15**/18.91 | **5.49**/20.29 | **5.21**/23.35 | **5.37**/20.08 | **5.06**/20.99 |  |
| D-glucose (3 g L^-1^) | **4.50**/nt | **4.44**/38.17 | **4.47**/38.92 | **4.19**/31.49 | **4.32**/34.84 | **4.23**/32.54 | **4.32**/35.64 | **4.33**/39.80 | **4.33**/30.12 | **4.13**/38.55 |  |
| D-glucose (5 g L^-1^) | **3.98**/nt | **4.00**/51.03 | **3.93**/55.49 | **4.16**/33.73 | **3.97**/43.96 | **3.96**/35.85 | **3.86**/48.14 | **3.91**/53.06 | **3.89**/41.16 | **3.98**/43.33 |  |
| D-glucose (10 g L^-1^) | **3.85**/nt | **3.88**/58.64 | **3.83**/49.89 | **4.10**/33.37 | **3.98**/42.40 | **3.92**/39.78 | **3.76**/50.47 | **3.77**/53.37 | **3.76**/46.04 | **3.99**/38.63 |  |
| D-glucose (1 g L^-1^) + D-mannose (2 g L^-1^) | **4.24**/nt | **4.36**/40.54 | **4.34**/39.21 | **4.17**/33.00 | **4.23**/36.64 | **4.19**/36.19 | **4.24**/37.10 | **4.23**/39.10 | **4.24**/30.97 | **4.23**/37.77 |  |
| D-glucose (1 g L^-1^) + D-mannose (4 g L^-1^) | **3.52**/nt | **3.91**/54.35 | **3.13**/50.07 | **4.17**/31.47 | **3.99**/43.33 | **4.02**/34.10 | **3.84**/52.81 | **3.99**/46.97 | **4.19**/36.61 | **4.24**/35.43 |  |

Footnotes: ^T^ type strain, ^G^ strain with *cyl* operon and prediction to produce granadaene pigment, nt – not tested.
